# An asymmetric bulge enhances artificial microRNA‐mediated virus resistance

**DOI:** 10.1111/pbi.13250

**Published:** 2019-09-18

**Authors:** Hui Zhang, Hui Feng, Xu Lu, Chaofan Wang, Wanneng Yang, Feng Li

**Affiliations:** ^1^ Key Laboratory of Horticulture Biology Ministry of Education College of Horticulture and Forestry Sciences Huazhong Agricultural University Wuhan Hubei China; ^2^ College of Informatics Huazhong Agricultural University Wuhan Hubei China; ^3^ College of Plant Sciences Huazhong Agricultural University Wuhan Hubei China; ^4^ College of Engineering Huazhong Agricultural University Wuhan Hubei China

**Keywords:** asymmetric bulge, P19, tomato bush stunt virus, artificial miRNA, viral resistance, crop protection

RNA silencing is a host defence mechanism against viruses in plants (Ding and Voinnet, 2007). As a counter defence mechanism, viruses have adapted ways to overcome the host RNA silencing pathway, such as viral suppressors of RNA silencing (VSR) (Incarbone and Dunoyer, 2013). Artificial miRNAs (amiRNAs) have been widely used as antiviral agents in plants as reported by Carbonell *et al*. (2019); Khalid *et al*. (2017); and Kis *et al*. (2016). However, the antiviral efficiency of amiRNAs has been countered by VSR. Some viral suppressors are capable of inhibiting miRNA function by binding to the miRNA duplex. For example, the P19 protein of tomato bush stunt virus (TBSV) forms a dimer and sequesters miRNAs by encasing the miRNA duplex (Vargason *et al*., 2003). According to structural modelling, an asymmetric bulge (AB) in the duplex region of miRNA precursors forms a protrusion on the outside of the RNA helices formed by hybridizations at the duplex region (Manavella *et al*., 2012). We hypothesize that AB in the miRNA precursor will affect the interaction between P19 and the miRNA/miRNA* duplex, thus improving the TBSV resistance abilities of amiRNAs.

To test our hypothesis, we generated twenty dual amiRNA clusters. Each amiRNA cluster contained two miRNA precursors. One precursor encoded an amiRNA targeting green florescence protein (MIR‐GFP or miRGFP, Figure 1a) with AB at various positions, and the other encoded a 21‐nt amiRNA targeting β‐glucuronidase (MIR‐GUS or miRGUS). A transient expression assay was conducted to screen for MIR‐GFPs that could silence GFP and resist P19 suppression. Most MIR‐GFPs were similar to S10B1 and M14B1, which could efficiently silence GFP expression when co‐expressed with 35S::GFP and EV, while the silencing was efficiently inhibited by co‐expressing 35S::P19 (Figure 1b, top two rows). Interestingly, S07B1 and M14B2 were found to efficiently silence GFP expression similar to other MIR‐GFPs when co‐expressed with 35S::GFP and EV (Figure 1b, left), but their silencing of GFP expression remained largely effective in the presence of 35S::P19, as shown by the much weaker green florescence signal (Figure 1b, comparing bottom two vs top two rows on the left). Western blotting was performed to further examine the GFP protein levels in leaves co‐expressing 35S::P19 and MIR‐GFPs. Consistent with the GFP imaging results, GFP protein levels were high in leaves expressing S10B1 and M14B1 but low in leaves expressing S07B1 and M14B2, while the control neomycin phosphotransferase II (NPT II) protein expressed from the 35S::GFP construct accumulated at similar levels in all samples (Figure 1c). To test whether ABs in these miRGFPs interfered with P19 binding, co‐immunoprecipitation experiments were performed using samples co‐expressing 35S::P19 and various MIR‐GFPs. As expected, miRGFP and miRGUS signals of the expected length and P19 protein were detected in P19/MIR‐GFP‐expressing samples but not in the EV control, while the endogenous miR166 control was detected in all input samples (Figure 1d, lanes 2‐5 vs. 1). Co‐IP experiments showed that miRGFP of 21 or 22 nt was detected in co‐IP products derived from samples expressing P19 plus S10B1 or M14B1, respectively, but not from samples expressing P19 plus either S07B1 or M14B2 (Figure 1d, lanes 7, 8 vs. 9, 10), while both controls miRGUS and miR166 were pulled down by P19 in all P19‐containing samples (Figure 1d, lanes 7–10). These results showed that P19 binding to S07B1 and M14B2 was greatly reduced, which was consistent with the resistance of these two miRGFPs to inhibition by P19.

**Figure 1:**
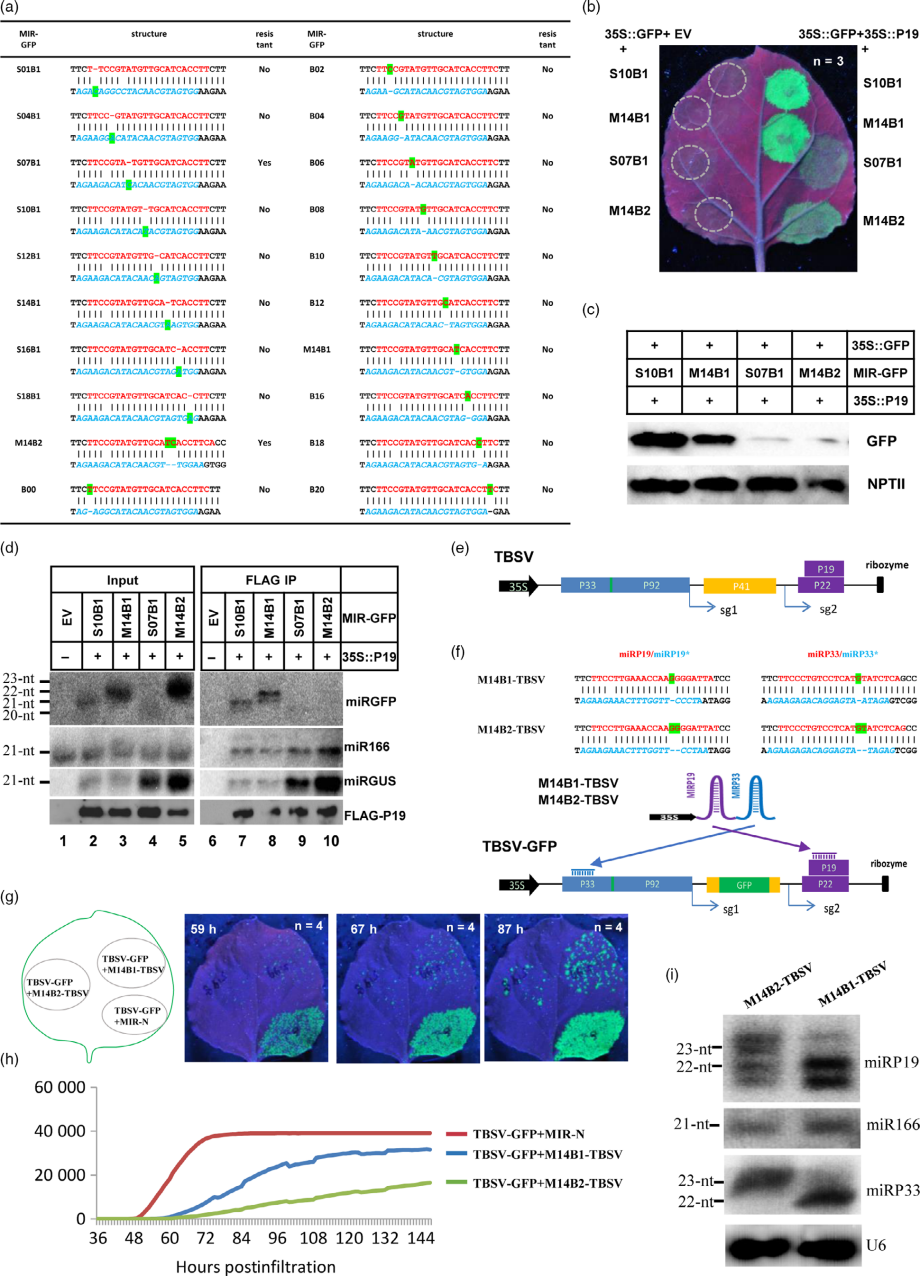
An asymmetric bulge in the miRNA/miRNA* duplex improved amiRNA‐mediated virus resistance. (a) Summary of MIR‐GFP structures and resistance to P19 inhibition. (b) S07B1 and M14B2‐mediated GFP silencing was resistant to P19 suppression. On the left, leaf patches were co‐infiltrated with 35S::GFP, EV and the indicated MIR‐GFP constructs. On the right, leaf patches were co‐infiltrated with 35S::GFP, 35S::P19 and the indicated MIR‐GFP constructs. Green florescence was imaged under a UV light at four days postinfiltration. The experiment was repeated three times. (c) Western blot analysis of GFP and NPTII protein levels in the samples shown on the right side of b. The genes expressed in each sample are indicated above each lane. The protein detected by primary antibody is indicated on the right side of each Western blot image. The experiment was repeated two times. (d) Northern blot detection of small RNAs (miRGFP, miR166 and miRGUS) and Western blot detection of P19 protein. Input RNA and protein samples were analysed on the left panel, and co‐immunoprecipitation products were analysed on the right panel. The experiment was repeated two times. (e) Design of TBSV infectious clones. (f) Sequences of anti‐TBSV AMIR clusters and genome structure of the TBSV‐GFP infectious clone. Top two rows include the miRNA (red) and miRNA* (blue) sequences. The AB is highlighted in green. The 35S promoter and ribozyme are shown as a black arrow and a vertical bar, respectively. The viral ORF is shown as a box with different colours. (g) Arrangement of infiltration patches in infiltrated leaves and automated imaging of viral replication in plants. Representative images showing the green fluorescence signal from GFP protein expressed by TBSV‐GFP at the indicated time points. The experiment was repeated four times. (h) Representative profile of the GFP signal during the observation time window. The *y*‐axis is in arbitrary florescence units, and the *x*‐axis is hours postinfiltration. The experiment was repeated four times. (i) Northern blot analysis of miRP19 and miRP33 expressed from two artificial miRNA clusters (indicated above each lane). The experiment was repeated two times. All repeated experiments showed similar results, and a representative result was presented in b–d and g–i.

Next, we tested whether M14B2 could confer better resistance in the context of virus infection. For this, a TBSV infectious clone was made by placing full‐length TBSV cDNA between the 35S promoter and a ribozyme (Figure 1e). For easy monitoring of TBSV replication in plant leaves, part of the viral coat protein sequence was replaced with GFP sequence to generate a TBSV‐GFP replicon, which replicated locally efficiently (Figure 1f and g). We also generated M14B1‐ and M14B2‐based AMIR clusters M14B1‐TBSV and M14B2‐TBSV, respectively, expressing two amiRNAs targeting viral P33 and P19 (Figure 1f and g), as well as a control AMIR not targeting TBSV (MIR‐N). TBSV‐GFP was co‐expressed with MIR‐N, M14B1‐TBSV or M14B2‐TBSV in different areas of the same leaves by agroinfiltration (Figure 1g), and GFP expression was monitored by a real‐time imaging system. The area of green florescence in each infiltrated patch was calculated at each time point and plotted into growth curves for TBSV‐GFP in each area (Figure 1h). The results showed that the GFP area quickly reached a maximum level producing an ‘S’ curve in the TBSV‐GFP and MIR‐N co‐infiltrated areas (Figure 1h). In contrast, the increase in the GFP area was significantly reduced when TBSV‐GFP was co‐expressed with M14B1‐TBSV or M14B2‐TBSV, and M14B2‐TBSV imposed a clearly stronger inhibition (Figure 1h). To rule out a potential dosage effect of amiRNA levels, northern blot was conducted to compare amiRNA accumulation from M14B2‐TBSV and M14B1‐TBSV, and the result showed that amiRNA accumulated at a lower level in the M14B2‐TBSV‐expressing site than in the M14B1‐TBSV‐expressing site (Figure 1i). These results demonstrated that the P19‐resistant M14B2‐TBSV could mount a more effective antiviral defence against TBSV compared with that of M14B1‐TBSV, which is nonresistant to P19.

Previous studies have shown that P19 has a lower affinity for a miR171 duplex with mismatches and for 22‐nt miR168 isomers with ABs, which explains the preference of P19 for binding to viral siRNAs during infection and the persistence of miR168‐mediated repression of its target AGO1 in the presence of P19 (Iki *et al*., 2018; Kontra *et al*., 2016). Here, we showed that ABs in S10B1 and M14B2 also reduced P19 binding and, more importantly, could be applied to improve amiRNA‐mediated TBSV resistance. One major concern about amiRNA‐mediated antiviral resistance is that viruses may mutate amiRNA target sites to evade targeting by the amiRNAs and gradually overcome the effect of the amiRNAs by inhibiting their activity with silencing suppressors. Exploiting miRNA structure features to generate amiRNAs that evade suppressor activity in conjunction with targeting multiple conserved sites in the viral genome provides an opportunity to further improve amiRNA‐mediated viral resistance. In addition to TBSV P19, many plant and animal viruses encode dsRNA binding proteins to counteract host defences mediated by RNA silencing. For example, potyvirus HcPro, tobamovirus replicase, cucumovirus 2b, nodavirus B2, vaccinia virus E3L and influenza virus NS1 all suppress RNA silencing by binding to dsRNA (Li and Ding, 2006). Thus, ABs and other miRNA structural features may have a broader application in the improvement of antiviral silencing technology against those viruses.

## Author Contributions

FL, HZ and WY planned and designed the research. HZ, HF, XL and CW performed the experiments and analysed the data. FL and HZ wrote the manuscript.

## Conflicts of interest

The authors declare no conflict of interest.
